# QRS Complex Enlargement as a Predictor of Ventricular Arrhythmias in Patients Affected by Surgically Treated Tetralogy of Fallot: A Comprehensive Literature Review and Historical Overview

**DOI:** 10.1155/2013/782508

**Published:** 2013-02-20

**Authors:** Pier Paolo Bassareo, Giuseppe Mercuro

**Affiliations:** Department of Medical Sciences “M. Aresu”, University of Cagliari, Policlinico Universitario, S.S. 554, bivio di Sestu, Monserrato, 09042 Cagliari, Italy

## Abstract

Tetralogy of Fallot (TOF) is a congenital heart disease frequently treated by surgical repair to relieve symptoms and improve survival. However, despite the performing of an optimal surgical repair, TOF patients are at times characterized by a poor long-term survival rate, likely due to cardiac causes such as ventricular arrhythmias, with subsequent sudden death. In the 80s it was irrefutably demonstrated that QRS prolongation ≥180 msec at basal electrocardiogram is a strong predictor for refining risk stratification for ventricular tachycardia in these patients. The aim of this research was to undertake a review of all studies conducted to assess the impact of QRS duration on the development of life-threatening ventricular arrhythmias in repaired TOF subjects.

## 1. Introduction

Tetralogy of Fallot (TOF) is a congenital heart disease characterized by a large ventricular septal defect, an aorta that overrides the left and right ventricles, obstruction of the right ventricular outflow tract, and right ventricle hypertrophy. Surgical repair of this pathology is recommended to relieve symptoms and to improve survival. However, despite the performing of an optimal surgical repair, TOF patients may feature a poor long-term survival rate, likely due to cardiac causes such as ventricular arrhythmias. The pathogenesis of this kind of tachycardia, thought to account for the majority of cases of sudden unexpected death in operated TOF patients, remains to be fully elucidated [1–3]. Consequently, life-threatening ventricular arrhythmia and sudden death continue to represent serious late complications following TOF repair. Several risk factors have been identified in predicting TOF patients at major risk of sudden death: severity of initial presentation of the disease, preoperative polycythemia, delay in surgical correction of the lesion, elevated left ventricular end-diastolic pressure, initial Blalock-Taussig shunt placement, ventriculotomy, and ventricular arrhythmias [4, 5]. The aim of this work was to undertake a paper of all studies conducted to assess the impact of QRS duration on the development of life-threatening ventricular arrhythmias in repaired TOF subjects. QRS complex corresponds to the depolarization of the right and left ventricles. Duration of the complex may be calculated simply by means of an electrocardiogram, an easy, economical, and noninvasive investigation that is usually readily available in an outpatient setting. This paper should provide help to primary care practitioners in identifying TOF patients at increased risk of developing ventricular arrhythmias and sudden death, in making appropriate and timely referrals, and in educating patients and their families.

## 2. Search Strategy

A Pubmed/Medline search was conducted using the MeSH terms: Fallot, QRS, mechanoelectrical interaction, ventricular tachycardia, arrhythmia, sudden death, pulmonary insufficiency, and combinations of the terms. Articles identified in this manner were retrieved and reference lists searched for additional relevant articles. The search was limited to English-language publications, and no other restrictions were applied. The Pubmed/Medline database was searched from its inception to November 2012.

## 3. Results

**Figure 1:**
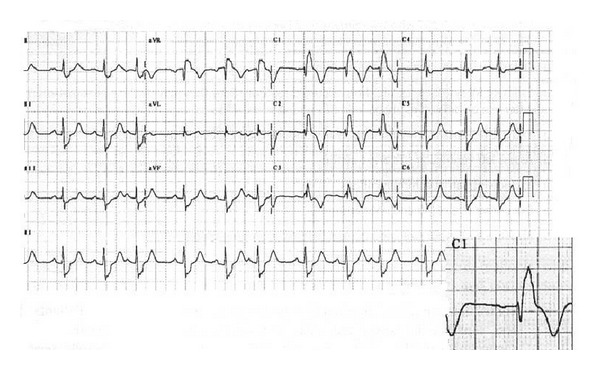
Right bundle branch with enlarged QRS complex (>180 msec) in a patient surgically treated for Tetralogy of Fallot.

A total of approximately one hundred and fifty studies focusing on this important issue were obtained, the most important of which are reported in this paper (Table 1). Since the 1970s and 1980s, several studies have been performed on the ECG findings of TOF patients, to ascertain the potential presence of signs capable of predicting the possible development of malignant arrhythmias in these patients. In 1984, for the first time, an important report published on the British Heart Journal showed that among patients with identical QRS morphology, life threatening ventricular tachycardias were more frequent in those with longer QRS prolongation (Figure 1) [6]. Taken together, all subsequent studies demonstrated how in patients previously treated by surgery for TOF, QRS prolongation predicts malignant ventricular arrhythmias, with QRS complex duration on surface ECG being the most sensitive predictor for development of a sustained monomorphic ventricular tachycardia [7]. Specifically, a QRS prolongation ≥180 msec is a predictor for late sudden death. Even following an initially good postsurgical outcome in TOF, QRS prolongation may progressively occur in later years. Accordingly, regular medical checkups—including electrocardiogram—are of fundamental importance following surgical correction for TOF [8]. It should however be underlined that particular accuracy is required in calculating QRS complex duration at ECG. Indeed, considerable controversy has arisen as to the most appropriate means of QRS measurement, with a study being undertaken to assess the accuracy and reproducibility of manual measurement of the QRS complex in standard electrocardiograms of patients with right bundle branch block, who had undergone radical repairs for TOF. The results were compared with computer readings, and significant differences thus detected in the measurement of QRS in the same ECG calculated twice by the same observer (with an absolute variation up to 50 msec), between different observers (*P* = 0.037) and measured manually or by computerized means (*P* = 0.019). The width of QRS did not influence measurements, as the largest intraobserver variation (50 msec) was observed for relatively wide complex (median value between the two measurements: 155 msec) and the largest interobserver (60 msec) for narrow complex (median value between the five measurements: 110 ms). QRS morphology appeared to influence the measurements, as the intra- and inter-observer variations were more consistent in the presence of notching, slurring, and terminal slow vectors. The results obtained underline the difficulty of obtaining QRS measurement both for the operator and in view of the influence produced by the presence of conduction abnormalities, thus reducing the accuracy and reproducibility of the findings [9].

## 4. QRS Duration and Right Ventricle

An association has been reported between ventricular enlargement secondary to pulmonary regurgitation in repaired TOF patients and a prolonged QRS duration at surface ECG [1]. However, the study concerned was based on the assumption that an increased cardiothoracic ratio at plain chest X-ray predominantly reflected right ventricular size. Residual shunts at ventricular level, tricuspid regurgitation, left ventricular dilatation, and the presence of pulmonary regurgitation may all result in an increased cardiothoracic ratio at chest X-ray. By combining ECG analysis with three-dimensional echocardiography technique in measuring right ventricular volumes, the hypothesis of a mechano-electrical interaction has been confirmed. These findings thus demonstrated how right ventricle enlargement is responsible for augmented QRS duration at ECG registration [10]. Right ventricle enlargement with subsequent wall-motion abnormalities is a common finding late after TOF repair and is associated with repolarization-depolarization abnormalities. These data further underscore the likelihood of a mechano-electrical interaction playing an important role in the pathogenesis of right ventricular disease  in  these  patients  [11]. Additional factors, in particular age and duration of followup, may also play a pivotal role. A recent study investigated the clinical profile and surgical outcomes of a TOF population. Major morbidities before cardiac repair included atrial arrhythmias (1.1%) and ventricular arrhythmias (3.9%). Ventricular arrhythmias occurred mainly after the age of 20years, with a preoperative QRS duration increment of 0.6 msec/year. Freedom from ventricular arrhythmia 30years after repair was observed in 84.1% of subjects and was associated with a final QRS exceeding 160 msec [12]. 

However, the major determinant of QRS prolongation in TOF patients remains right ventricular dilatation, which is influenced by the severity of pulmonary valve regurgitation [10]. It is an acknowledged fact that pulmonary insufficiency predisposes to ventricular arrhythmias, presumably due to progressive enlargement and stretching of the right ventricle. In this respect, QRS duration is related to right ventricular size as estimated by magnetic resonance imaging [13].

Several other concomitant mechanical factors have been indicated in potentially contributing towards QRS complex duration; these include not only increased right ventricular volume, but also increases in left ventricular volume and in right and left ventricular wall mass [14]. Indeed, even abnormal left ventricular mechanics—in combination with right ventricular dysfunction—may explain the correlation between QRS duration and adverse arrhythmic events. In this respect, vector velocity echocardiographic imaging was used to assess longitudinal strain and intraventricular dyssynchrony. Prolonged QRS duration was associated with increased right ventricle and left ventricle dimensions (*P* = 0.01) and decreased function (right ventricular ejection fraction: *r* = −0.60, *P* < 0.001; left ventricular ejection fraction: *r* = −0.77, *P* < 0.001). In addition, prolonged QRS duration has been associated with heterogeneous ventricular mechanical activation and reduced strain in the lateral and septal left ventricle walls [15]. 

Even following successful pulmonary valve replacement, severe QRS prolongation and the absence of a reduction in QRS duration are major determinants of an adverse outcome during long-term followup of patients with TOF [16]. Several studies have shown how the occurrence of both ventricular tachycardia and sudden death remains unaltered even in the presence of an apparent reduction in pulmonary regurgitation and right ventricular dilation [17, 18]. To this regard, further studies reported how successful pulmonary valve replacement produced a beneficial effect on electrocardiographic indices of repolarization heterogeneity. Normal repolarization indices are associated with the absence of severe ventricular arrhythmias in postsurgery TOF [19]. In these studies, a successful pulmonary valve surgery resulted in stabilization of QRS duration and, particularly in conjunction with intraoperative cryoablation, in a decrease in the incidence of preexisting ventricular tachyarrhythmia. When possible, this combined approach should be used in patients late after repair of TOF [20, 21]. To further address the issue, a retrospective multicentric study was also undertaken to analyze the long-term course of QRS duration after pulmonary valve replacement in patients with a previous correction for TOF. Mixed linear regression model was used to analyse the course of QRS duration over time. A decrease in QRS duration directly after surgery was observed, followed by a steady increase, in patients with a preoperative QRS > 150 msec. The beneficial effect of pulmonary valve replacement on QRS duration was transient, although risk of developing ventricular arrhythmias after surgery was substantial in the presence of a preoperative QRS of > or =180 msec [22]. An important study demonstrated the presence of ventricular fibrosis—detected by late gadolinium enhancement cardiovascular magnetic resonance—in adults with repaired TOF. Fibrosis was related to adverse markers of outcome, including ventricular tachycardia. Specifically, the above technique was performed to analyze right and left ventricles of 92 adult patients who had undergone TOF repair. A marked fibrosis was observed in the right ventricle of all patients at surgical sites located in the outflow tract (99%), in ventricular septal defect patching (98%), in the inferior right ventricle insertion point (79%), and in the trabeculated myocardium (24%). In the left ventricle, fibrosis (53%) was located at the apex consistent with apical vent insertion (49%), in the inferior or lateral wall consistent with infarction (5%), or in other areas (8%). Patients with severe right ventricular fibrosis were older (38 versus 27 years, *P* < 0.001) and more symptomatic (38% versus 8% in New York Heart Association class II or greater, *P* = 0.001). In addition, these patients featured increased clinical ventricular arrhythmias (26% versus 10%, *P* = 0.039). Nonapical left ventricular fibrosis also correlated with ventricular arrhythmias. Again, in a multivariate model, right ventricular fibrosis remained a predictor of ventricular arrhythmias. TOF patients with more diffuse ventricular fibrosis were those with QRS prolongation >180 msec [23]. 

Postoperative factors that may lead to prolongation of the QRS in this setting include valve deterioration with stenosis other than regurgitation [24]. Previously published studies suggest that residual right ventricular outflow tract gradients may be highly significant contributors to QRS prolongation. However, different opinions exist about this issue [25, 26]. During intrinsic rhythm, non-invasive mapping demonstrated delayed activation of the right compared with the left ventricle in TOF patients, with the greatest activation delay noted near the infundibulum. Thus, prevention and treatment of mechanical asynchrony and malignant arrhythmia should focus on the right ventricle infundibulum. It is a probable conclusion that patients after repair of TOF could be candidates for cardiac resynchronization therapy, biventricular pacing resulting more effective than right ventricular pacing in improving tolerance to exercise and lowering NYHA functional class [27]. Today nevertheless, a right ventricular approach is the most widely used in this selected group of TOF patients. It is well known that right ventricular pacing increases QRS duration in this population. Thus, an attempt was made to counteract increased QRS duration by means of optimization of the atrioventricular interval in a subset of repaired TOF patients, potentially affording right ventricular resynchronization in patients with baseline right bundle branch block. In short, this may imply that to achieve an effective pacing therapy, it may be necessary to establish site-specific placement of pacing leads and precise pacemaker programming [28]. 

## 5. QRS Duration Coupled with Other Electrocardiographic Parameters

Early detection of ventricular arrhythmias following TOF surgery is fundamental in contributing towards decreasing morbidity and mortality. The frequency range of the standard electrocardiogram signal is between 0.05 and 100 Hertz, although higher frequencies may at times be detected. Using high-resolution technology, the peak amplitudes of these high-frequency components within the QRS complex can be recorded and analyzed. To this regard, the relationship between ventricular late potentials, ventricular arrhythmias, and right ventricular systolic pressure was studied in 22 patients who had undergone TOF repair (mean followup 40.1 +/−33.5 months). Holter ECG monitoring and signal-averaged electrocardiograms were performed. The studied parameters of this technique included duration of filtered QRS, duration of terminal QRS below 40 *μ*V, and root mean square amplitude of the terminal 40 msec. Cardiac catheterization was performed on 19 of the patients studied. Eighteen healthy volunteers were enrolled as controls. Ventricular arrhythmias were found in 13 patients; right ventricular systolic hypertension was found in 1 patient. No significant residual ventricular septal defects were detected. Eight patients had ventricular late potentials, whilst right ventricular systolic pressure did not differ significantly between patients with or without late potentials. Significant differences were detected between patients with ventricular arrhythmias and healthy volunteers; filtered QRS duration was significantly longer in patients with ventricular arrhythmias. Accordingly, signal-averaged electrocardiograms may prove beneficial in determining ventricular arrhythmia risk in TOF patients postoperatively [29]. Specifically, negative signal-averaged electrocardiograms results connote the absence of a reentrant substrate and, therefore, the absence of risk for reentrant monomorphic ventricular tachycardia, whereas positive signal-averaged electrocardiograms results suggest the presence of a slow conduction substrate and potential risk for monomorphic ventricular tachycardia [30]. 

The findings of this study may provide guidance for use in risk stratification and therapeutic interventions in repaired TOF patients [31]. A study has likewise been conducted to compare QRS duration at standard ECG and signal-averaging ECG for arrhythmic risk stratification subsequent to surgical repair of TOF. The aim of the study was to identify patients who had undergone surgery for TOF who were at higher risk of sudden death by means of signal-averaging ECG. Sixty-six patients (mean age 26 +/− 10 years) were studied (17.7 +/− 5.8 years after total correction for TOF) by means of standard ECG, 24-hour Holter recordings, signal-averaging ECG, and echocardiography. The following variables were measured: standard QRS duration, filtered QRS duration, high-frequency and low-amplitude signal duration, root mean square of the mean voltage in the terminal portion of filtered QRS, left and right end-diastolic volumes, and ejection fractions. During a mean follow-up period of 7.3 +/− 3.1 years, 12 patients featured episodes of sustained ventricular tachycardia and two died suddenly. All patients were affected by complete right bundle branch block. Patients with ventricular tachycardia were characterized by a significant longer filtered QRS duration at all finger settings. On the contrary, no differences were revealed in standard QRS duration in patients with or without ventricular tachycardia. On multivariate analysis, left ventricular ejection fraction and filtered QRS duration were independent predictors for ventricular tachycardia. In short, a longer filtered QRS duration is associated with an increased risk of developing malignant ventricular arrhythmias in asymptomatic patients after total correction of TOF [32]. 

Microvolt T-wave alternans, another possible indicator of repolarization heterogeneity, has recently been investigated to assess its potential as a predictor of late ventricular arrhythmia in adults with TOF. A total of 101 repaired TOF patients (60.4% male) were studied in comparison with a control group of 103 age- and sex-matched subjects with normal hearts. Age at followup was 20.0 ± 10.6 years. Total surgical TOF repair (60.4% received a transannular right ventricular outflow patch) was performed at a mean age of 4.8 ± 5.8 years. After having excluded 11 patients with indeterminate data, the microvolt T-wave alternans values in 90 TOF patients revealed higher values than those of controls (25.1 ± 14.0 versus 17.6 ± 9.2 *μ*V, *P* < 0.001). The values obtained were correlated with the presence of severe pulmonary regurgitation (*P* = 0.006). Ten patients (9.9%) experienced late ventricular arrhythmic events and tended to have higher microvolt T wave alternans values than those lacking ventricular arrhythmias (34.0 ± 16.5 versus 24.2 ± 13.5, *P* = 0.053). Although microvolt T wave alternans per se was not superior to QRS duration alone in predicting late arrhythmia, positive and negative predictive values increased slightly after adding the microvolt T wave alternans to QRS duration.

Although sustained microvolt-level T wave alternans has been proposed as a marker of increased risk for malignant ventricular arrhythmia following repair of TOF, further studies are required to determine whether—alone or in combination with QRS enlargement—it may represent an increased risk for arrhythmia in this patient group [33].

Furthermore, increased QT and JT dispersions, combined with a QRS ≥ 180 ms, refine risk stratification for ventricular tachycardia in these patients, thus suggesting that both depolarization and repolarization abnormalities are associated with ventricular tachycardia after repaired TOF. To this concern, in a previous study QRS duration and QT/QRS/JT dispersion were measured manually from standard ECGs in 10 syncopal repaired TOF patients with QRS ≥ 180 ms and with documented ventricular tachycardia (group 1). They were compared with 9 repaired TOF patients with QRS ≥ 180 ms and no ventricular arrhythmias (group 2), with 40 repaired TOF patients having QRS < 180 ms and no clinical arrhythmias (group 3), and with 40 healthy subjects enrolled as controls (comprising 20 with right bundle branch block (group 4) and 20 with normal ECG patterns (group 5)). Mean QT dispersion in the TOF patients was greater than in the control group (*P* < 0.001). Significant differences were revealed in all measured parameters between groups 1 and 3, and more importantly between groups 1 and 2. QRS dispersion in group 1 also correlated with QRS duration, but not with JT dispersion [34]. However, a prolonged QRS duration alone in postoperative TOF with right bundle branch block is more predictive than QTc, JTc, or dispersion indexes in identifying vulnerability to ventricular arrhythmias in this population, while retaining high specificity [35]. 

However, even the latter ECG parameters may be influenced not only by right ventricular enlargement, but also by left ventricular volume and biventricular wall mass. Indeed, when assessing left and right ventricular size in repaired TOF patients by magnetic resonance imaging, and the amount of pulmonary regurgitation by velocity mapping magnetic resonance imaging, both biventricular volumes and right ventricular wall mass were seen to be increased. When QT, QRS, JT duration, and interlead dispersion markers were derived from a standard 12 lead ECG, significant differences were registered. In fact, mean QRS duration was significantly prolonged in TOF, as well as dispersion of QRS, QT interval, and JT interval. QRS duration correlated best with right ventricular mass (*r* = 0.55, *P* < 0.01). Therefore, in patients who have undergone surgical correction of TOF, all ECG predictors for ventricular arrhythmias are influenced by a series of mechanical factors which involve the two ventricles and which may occur simultaneously [14].

## 6. QRS Duration and Physical Exercise

The above stated electrocardiographic signs (QRS enlargement combined with QTc and JTc) continue to confirm their efficiency in predicting increased risk for ventricular arrhythmia even during physical exercise. In a study aimed at assessing proarrhythmogenic electrocardiographic changes during maximal physical exercise in patients operated for TOF, R-R duration; QRS, QT, and JT duration, and dispersions were assessed. ECG data were analyzed at rest, at 60% of peak exercise and at peak exercise.

Changes in ECG parameters during exercise were calculated and correlated to right ventricle volume, right ventricle ejection fraction, right ventricle wall mass, PR percentage, and VO_2_ max. Exercise ECG data from healthy controls were used as reference.

On exercise, mean QTc and JTc dispersions increased in TOF patients (*P* < 0.001), but not in controls. At peak exercise, JTc dispersion was larger in TOF patients than in healthy controls (*P* < 0.01). QTc was not modified with exercise in TOF patients (*P* = ns) and decreased in controls (*P* < 0.05). At all levels of exercise mean QTc, QRS duration, and QRS dispersion were larger in TOF patients (all *P* < 0.001). Significant associations were found for (1) a greater increase of JTc dispersion with a higher PR percentage, a larger right ventricle volume, a larger right ventricle wall-mass and (2) greater QTc increase with a larger right ventricular volume and reduced right ventricle ejection fraction. In conclusion, during physical exercise inhomogeneity of repolarisation, known to predispose to reentry ventricular arrhythmia, increased in repaired TOF. A greater inhomogeneity was found in the presence of more severe pulmonary regurgitation [36]. A temporal and regional variation in ventricular repolarization across the myocardium in patients with repaired TOF could represent a pathophysiological substrate for an increased cardiac electrical instability. The presence of negative prognostic factors, relating to surgical intervention or residual haemodynamic abnormalities, even when lacking a direct influence on the invariably present arrhythmic substrate, may “trigger” conditions underlying the development of ventricular arrhythmias [37].

Recently, a correlation between fragmented QRS and right ventricular fibrosis, detected by late gadolinium enhancement at cardiac magnetic resonance, has also been demonstrated in adult TOF patients [38]. 

Furthermore in adult patients with repaired TOF, QRS duration at rest seems to be a predictor of maximal exercise capacity, and changes in QRS duration are likewise associated with the latter. Thus, in a group of TOF patients, QRS duration in V_1_ (msec) was measured at rest, at maximal exercise (*W*_max⁡_, Watt), and at peak oxygen consumption (peak VO_2_, mL/min). Stroke volume was calculated from cardiac output, obtained by CO_2_ rebreathing. The study findings showed that in patients with QRS shortening, peak VO_2_ and exercise-induced increase in stroke volume were significantly higher than in patients with QRS prolongation. This study indicated that QRS shortening during exercise in TOF patients was related to a better exercise performance [39]. Another study showed how exercise stress induced biventricular mechanical dyssynchrony in children with TOF having no signs of dyssynchrony at rest. This issue however proved to be unrelated to QRS duration [40].

## 7. QRS at Electrophysiologic Study

Electrophysiological studies have shown that prolonged QRS duration at electrocardiogram is predictive not only of spontaneous adverse arrhythmic events, but even inducible ventricular tachycardia. To investigate this interesting issue, 135 survivors of TOF surgery were studied from 1984 to 1995. Age at surgery ranged from 34 days to 37 years (3.7 ± 3.9 years, median 2.5) and age at electrophysiological study was 1.4 to 43 years (9.7 ± 8.2 years, median 6.7). QRS duration was 80 to 240 msec (137 ± 29 msec). Sustained ventricular tachycardia was induced in 22 patients (monomorphic in seventeen). Induced sustained monomorphic tachycardia was related to QRS duration, right ventricular dimension, H-V interval, and presence of symptoms at univariate analysis. QRS duration was also related to induced sustained monomorphic ventricular tachycardia at multivariate analysis. QRS duration > or =180 msec was 35% sensitive and 97% specific for induced sustained monomorphic ventricular tachycardia at electrophysiological study. This relationship persisted even when analyzing solely asymptomatic patients. QRS duration > or =180 msec was 100% sensitive and 96% specific for detecting clinical ventricular tachycardia. In the opinion of the authors of the reported paper, the finding of prolonged QRS duration should suggest the need for further testing to determine the risk of adverse arrhythmic events in patients after repair of TOF, even when asymptomatic [41]. The status of the sympathetic nervous system in postoperative TOF patients, known to play an important role in other patients at risk for ventricular arrhythmias, remained unknown until an important study was performed to define its role in determining ventricular electrical instability. After i.v. injection of metaiodobenzylguanidine, a reduction in vagal control and sympathetic dominance was demonstrated in all patients compared with a healthy control group, as testified by the increase in heart rate variability. The uptake of metaiodobenzylguanidine was significantly reduced in TOF patients at risk of ventricular tachycardia or fibrillation. In addition, tomographic imaging techniques revealed a decrease in the number of nerve endings in the right and left ventricular walls, together with an inhomogeneous distribution of the adrenergic nervous system. The positive correlation between myocardial uptake of metaiodobenzylguanidine, standard deviation of all adjacent intervals between normal beats, and QRS dispersion confirmed the appropriateness of analyzing adrenergic nervous system to stratify TOF patients at risk of life-threatening arrhythmias [42]. Other markers of global impairment in the regulation of the autonomic nervous system late after repair of TOF include a marked reduction of baroreflex sensitivity and heart rate variability. These would appear to be related to previous surgical intervention/s, timing, and current right and left-sided hemodynamics. Reduced heart rate variability was also found to be related to the above stated markers of sustained ventricular tachycardia and sudden cardiac death (including QRS enlargement), thus suggesting possible common pathogenic mechanisms. Further studies are required to examine the prognostic significance of these impaired markers [43]. Interracial differences have also been reported to influence the development of ventricular arrhythmias. In a nationwide multicenter Japanese study, the prevalence of serious arrhythmias was low among TOF patients compared to the results obtained in Western countries. It has been hypothesized that this excellent result could be related to the narrow QRS after surgery. In fact, 60% of the subjects had QRS duration <120 msec [44]. 

## 8. Therapeutic Options

The use of antiarrhythmic drugs (Ib agents, beta blockers, and amiodarone), widely prescribed in the past, is now limited to the following conditions: (1) patients with high grade ectopic ventricular activity and poor haemodynamic conditions who are not candidates for corrective surgery and do not have sufficiently severe symptoms to justify defibrillator implantation; (2) patients with a defibrillator and frequent episodes of ventricular tachycardia, in order to lower the number of shocks; (3) patients with well-tolerated ventricular tachycardia and good haemodynamic conditions, who are unwilling to undergo ablation or in whom ablation has been unsuccessful and the efficacy of medications has been documented by an electrophysiological study [45].

Implantable cardioverter defibrillators are an important adjunct in the management of repaired TOF with QRS > 180 msec. Nevertheless, as malignant arrhythmias may even be manifested in patients with no residual lesions, QRS prolongation, and ventricular dysfunction, the identification of subjects who would benefit from implantable cardioverter defibrillators remains a clinical challenge [46]. Another therapeutic option in this setting is represented by the ablation of ventricular arrhythmias. Lastly, cardiac resynchronization therapy, with particularly biventricular pacing, has proven to be particularly promising, although further in-depth studies are required prior to widespread implementation [47]. 

## 9. Conclusions

Unequivocal findings have demonstrated that in TOF patients a QRS prolongation ≥180 msec is a predictor for late sudden death. These results further underline the involvement of depolarization abnormalities in arrhythmogenesis in postrepair Fallot patients [48]. This issue has indeed been observed both in patients receiving transannular patch, as well as in those who received no patch or a pulmonary homograft [49]. Furthermore additional ECG parameters, including increased QT, signal-averaging ECG, microvolt T wave alternans, and JT dispersions, combined with a QRS ≥ 180 ms, refine risk stratification for ventricular tachycardia in these patients, thus suggesting that both depolarization and repolarization abnormalities are associated with ventricular tachycardia after surgical repair of TOF. Additionally, the strong relationship between QRS prolongation and marked right ventricular enlargement at chest X-ray, echocardiography, and cardiac magnetic resonance has also been demonstrated, implicating a mechano-electrical interaction in progressive QRS enlargement. Indeed, pulmonary insufficiency predisposes to ventricular arrhythmias, presumably due to progressive enlargement and stretching of the right ventricle [50, 51]. Furthermore, electrophysiological evidence underlines how sustained ventricular tachycardia in this setting is frequently the outcome of a reentry circuit. Tachycardia may be resolved by implantation of cardioverter defibrillator or eradicated by intraoperative or postoperative radiofrequency catheter ablation.

## Figures and Tables

**Table 1:** Different types of studies performed to investigate the influence of QRS duration on the development of ventricular arrhythmias in patients who had undergone surgery for Tetralogy of Fallot.

Research area	Ref.
First report	[6]
Reviews	[1–3, 7, 8, 48]
Risk factors	[4, 5, 49]
QRS measurement modality	[9]
QRS duration and right ventricle size	[1, 10–28, 50, 51]
QRS duration coupled with other ECG parameters	[29–35]
Influence of physical exercise on QRS duration	[36–40]
QRS and electrophysiological study	[41–44]
Therapeutic options	[45–47]
